# Inertial Aided Cycle Slip Detection and Identification for Integrated PPP GPS and INS

**DOI:** 10.3390/s121114344

**Published:** 2012-10-25

**Authors:** Shuang Du, Yang Gao

**Affiliations:** Department of Geomatics Engineering, the University of Calgary, 2500 University Drive NW, Calgary, AB T2N 1N4, Canada; E-Mail: ygao@ucalgary.ca

**Keywords:** cycle slips, detection and identification, inertial sensors, precise point positioning

## Abstract

The recently developed integrated Precise Point Positioning (PPP) GPS/INS system can be useful to many applications, such as UAV navigation systems, land vehicle/machine automation and mobile mapping systems. Since carrier phase measurements are the primary observables in PPP GPS, cycle slips, which often occur due to high dynamics, signal obstructions and low satellite elevation, must be detected and repaired in order to ensure the navigation performance. In this research, a new algorithm of cycle slip detection and identification has been developed. With the aiding from INS, the proposed method jointly uses WL and EWL phase combinations to uniquely determine cycle slips in the L1 and L2 frequencies. To verify the efficiency of the algorithm, both tactical-grade and consumer-grade IMUs are tested by using a real dataset collected from two field tests. The results indicate that the proposed algorithm can efficiently detect and identify the cycle slips and subsequently improve the navigation performance of the integrated system.

## 1. Introduction

The advent of the Precise Point Positioning (PPP) GPS technique, capable of providing centimeter to decimeter positioning accuracy using a single GPS receiver, makes it possible to develop high precision GPS/INS integrated system without the need to set up base receiver stations [1]. Such PPP GPS/INS integrated system will be of value to applications where base receiver stations are difficult to setup in the field. Some examples of these applications are disaster assessment and management, life search and rescue, environmental monitoring and resources exploration, which are operated in either remote regions or challenging environments [2,3].

Although PPP has been widely used for different applications, it is still vulnerable to signal tracking interruptions, which cause cycle slips. The PPP usually requires a convergence time (typically tens of minutes) to achieve the centimeter to decimeter level positioning accuracy due to the fact that the ambiguities in the carrier phase measurements need a time period to converge to their true values. In case of satellite signal loss, the cycle slips will lead to biased solutions and PPP usually needs up to tens of minutes to re-converge to the centimeter-to-decimeter positioning accuracy. For PPP or integrated PPP GPS/INS to maintain high level positioning accuracy, cycle slips caused by the loss of signals must be detected and repaired.

Some general methods of cycle slip detection and identification have been summarized in the GPS literature [4–6]. Some examples are phase-code comparison, phase-phase ionospheric residual, differential phases of time. The phase-minus-code comparison method is one of the simplest methods to detect cycle slips; however it is insensitive to small cycle slips (e.g., 1–2 cycles) due to the high level noise and multipath of the code measurements. The phase-minus-phase ionospheric residual method is unable to detect the cycle slips which occur frequently, though it is very effective in detecting cycle slips, and is insensitive to the ionosphere variation. The differential phases of time requires polynomial fitting to interpolate or extrapolate the data at the check epoch, however it is still not effective for detecting and identifying small cycle slips.

There is a considerable volume of research on the topic of cycle slip detection and identification in differential GPS (DGPS), which has been extensively accepted by the GPS community due to its high level positioning accuracy. A wide-lane based cycle slip detection and ambiguity resolution algorithm for DGPS is developed by [7]. Some other methods include the automated cycle slip detection and repair method for kinematic DGPS [8] and a real-time cycle slip repair method proposed by [9]. Unlike PPP, DGPS employs one rover station receiver and one base station receiver. The measurement errors for both the rover station and base station receivers are highly correlated (if the baseline is not too long), so the observation differencing algorithm can effectively remove the measurement errors and this eventually will result in high quality solutions. The cycle slip detection and repair methods developed for the DGPS all require at least two GPS receivers to perform the observation differentiating algorithm; therefore they are not suitable for the cycle slip detection and identification in PPP or integrated PPP/INS systems, which only employ a single dual-frequency GPS receiver.

Due to the lack of the base station receiver, the measurement error mitigation in PPP relies on precise satellite orbit and clock corrections, as well as additional error modeling and estimation to obtain high accuracy solutions. However the measurement errors cannot be completely removed and the magnitude of the residual errors is much larger compared to the errors in DGPS, so the cycle slips will be detected and identified by the presence of the residual measurement errors in PPP, which makes the task more challenging. A method is developed based on the time-differenced code and phase measurements and using a Least-Square adjustment to estimate the size of the cycle slips [10]. This method has two shortcomings: (1) the use of code measurements may degrade the overall performance due to the their high level noise and multipath and (2) the employment of an adjustment procedure makes the data processing more complex. Some methods using triple frequency GPS signals to detect cycle slips have been proposed, such as [11–13], however the use of dual-frequency GPS receivers still prevails in applications, as not all GPS satellites transmit data in triple frequencies. An automated cycle slip detection and repair method, which is based on ionospheric total electron contents (TEC) rate (TECR) and Melbourne–Wübbena wide-lane (MWWL) linear combination to determine the cycle slip in both the L1 and L2 frequencies was developed by [14]. However, the limitation of this method is that the code measurements are used for MWWL generation and the noise and multipath of the code measurements are relatively larger than the wavelength of MWWL, which could potentially deteriorate cycle slip detection.

For the integrated GPS/INS system, the inertial information is frequently used to aid the cycle slip detection and identification. Some examples are [15–17]. However, these methods are designed specifically for DGPS, and they are not suitable for PPP/INS integrated systems. Moreover they use high-end IMUs (tactical-grade IMU) to ensure the quality of the inertial aiding for cycle slip detection and identification, which constrains significantly their feasibility in many applications due to the expensive cost of the INS system.

In this paper, we propose a new method of inertial aided cycle slip detection and identification for the PPP GPS/INS integrated system. The method jointly uses the wide-lane (WL) phase combination and extra wide-lane (EWL) phase combination to uniquely determine the cycle slips in the L1 and L2 frequencies [3,18]. Different from [14], to avoid the relatively high noise and multipath resulting from code measurements, the WL phase is generated only using phase measurements, and the INS solution is used to remove the range information from the WL phase to get the geometry-free combination (the details will be introduced in Section 3). The EWL phase is another linear combination of phase measurements, which has a wavelength of 183 cm. The generation of the EWL phase is similar to the WL phase, but using different linear coefficients. As mentioned before, the residual measurement errors make the cycle slip detection and identification more challenging in PPP GPS. In our method, the use of WL and EWL phases can enhance the efficiency and robustness of the cycle slip detection and identification even in the presence of residual measurement errors because of their much longer wavelength [3,18]. Both high-end IMU (tactical-grade) and low-cost MEMS IMU (consumer-grade) systems are tested in different field tests and the results indicate that the cycle slips can be detected and identified with very high confidence level using both grades of IMUs. During the field test, the GPS data rate is 1 Hz, which is very typical for GPS applications when integrating with INS for position and attitude determination, as the INS system needs frequent update information to calibrate the inertial sensor biases and to limit the INS error accumulation. Different from [15–17], the use of the INS system will not constrain the feasibility of our method to consumer applications, as the proposed method can be applied to low-cost MEMS IMU, which only cost a few dollars to a few hundred dollars. To our knowledge, this is the first cycle slip detection and identification method using inertial aiding for the PPP GPS/INS integrated system and also the first method that can be applied to low-cost MEMS IMUs.

The remainder of this paper is organized as follows: Section 2 introduces the PPP and the integrated PPP GPS/INS system; Section 3 provides details of the developed inertial aided cycle slip detection and identification algorithm; two field tests and their results are presented in Section 4, followed by the conclusions in Section 5.

## 2. Integrated PPP GPS/INS System

### 2.1. Precise Point Positioning GPS

PPP uses un-differenced code and phase measurements from a single dual-frequency GPS receiver, in addition to precise satellite orbit and clock data [3,19–21]. This technique does not suffer from the drawbacks of the conventional DGPS and is able to provide similar positioning accuracy comparable to DGPS without the need for a base station [22]. Precise satellite orbit and clock products are provided by several organizations, such as the International GPS Service (IGS) and Jet Propulsion Laboratory (JPL) [19–21]. The traditional measurement model for PPP includes the ionosphere-free code and phase measurements, which can be described by Equations (1) and (2), respectively [3,23–26]. The satellite orbit and clock errors are assumed to be removed by applying the precise satellite orbit and clock products:

(1) $$P_{IF}=\rho +\text{cdT}+d_{\text{trop}}+\varepsilon (P_{IF})$$

(2) $$\Phi_{IF}=\rho +\text{cdT}+N_{IF}\lambda_{IF}+d_{\text{trop}}+\varepsilon (\Phi_{IF})$$

where *P_IF_* and Φ*_IF_* are the ionosphere-free code and phase measurements in units of meters, respectively, *ρ* is the true range between the GPS receiver and the satellite in units of meter, c is the light speed in metre per second units, *dT* is the receiver clock offset in units of seconds, *N_IF_* and *λ_IF_* are the ionosphere-free ambiguity and wavelength, respectively, *d_trop_* is the tropospheric delay in meters, and *ε* is the measurement noise and multipath in the unit of metre. It is well known that the ionosphere-free ambiguities do not conserve the integer characteristics of ambiguities, so it is not practical to implement the cycle slip detection and identification on the ionosphere-free measurements. Instead, cycle slips will be detected and identified in both the L1 and L2 frequencies, respectively, and the method will be introduced in Section 3. Since the success of PPP significantly improves the operational flexibility and reduces the system cost, it increases the number of possible applications using GPS technology, such as geodetic survey, machine control and atmosphere sensing [26,27].

### 2.2. Integrated PPP GPS/INS System

The PPP GPS can be integrated with INS in both tightly and loosely coupled modes. In both cases, the inertial sensor errors can be continuously estimated whenever PPP GPS solutions are available. As a result the INS error accumulation can be bounded. On the other hand, the inertial sensors can bridge the navigation solution during PPP GPS outages. The obtained navigation solution from an integrated system would therefore outperform the standalone positioning solution. Although the cycle slip detection and identification algorithm proposed in this paper can be implemented in both tightly and loosely coupled systems, only the tightly coupled integrated PPP GPS/INS system is briefly introduced in this paper. The details of the loosely coupled approach can be found in [3].

**Figure 1.**
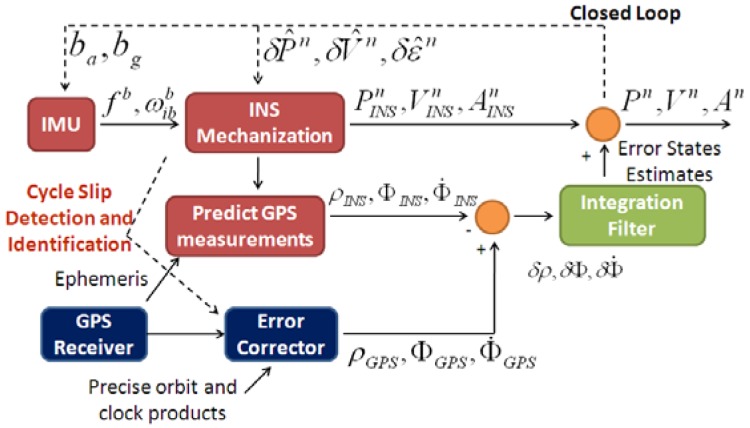
Tightly coupled PPP GPS/INS integrated system.

Illustrated in Figure 1 is the integration scheme of a tightly coupled PPP GPS/INS system. A single integration filter is used to fuse the PPP GPS and INS information. Given GPS satellites ephemeris data, the outputs of the position and velocity solutions from INS mechanization are used to predict the pseudorange, carrier phase and Doppler measurements. The error corrector in PPP GPS is used to correct the errors in the raw GPS measurements, such as the satellite orbit and clock errors. The ionospheric delay is eliminated by using the ionosphere-free linear combination of measurements from the L1 and L2 frequencies. The corrected PPP GPS measurements are then differenced with the INS-predicted measurements. Next the integration filter processes the residuals between PPP GPS and INS-predicted measurements to derive the INS error estimates. Finally, the obtained INS error estimates are feedback to INS mechanization using a closed loop approach. The outputs of INS mechanization are also used in the error corrector of PPP GPS to detect and identify any possible cycle slip and enhance the quality control of the integrated system.

Both high-end IMU and low-cost MEMS IMU can be used in the proposed integrated system. However as the performance and sensor error characteristics of the two types of IMUs are quite different, the Kalman filter design for each will be different too [28]. The differences are generally twofold. On the one hand, the parameters selected for error modeling of inertial sensor errors would be different for the two types of IMUs [29,30]. On the other hand, low-cost MEMS IMU normally feature significant turn-on biases and scale factors, which are usually ignored for the high-end IMUs. As a result, more error states are needed to augment the Kalman filter states for the low-cost MEMS IMU [3]. Details of the estimation approach for Kalman filter can be found in [31,32].

## 3. The Algorithm of Inertial Aided Cycle Slip Detection and Identification

The proposed inertial aided cycle slip detection and identification algorithm jointly uses the WL and EWL phases to determine cycle slips in both the L1 and L2 frequencies. It consists of three steps, namely, WL phase based cycle slip detection and identification, EWL phase based cycle slip detection and identification, and cycle slip identification in the L1 and L2 frequencies. They will be described in details in the following sections.

### 3.1. WL Phase Based Cycle Slip Detection and Identification

**Figure 2.**
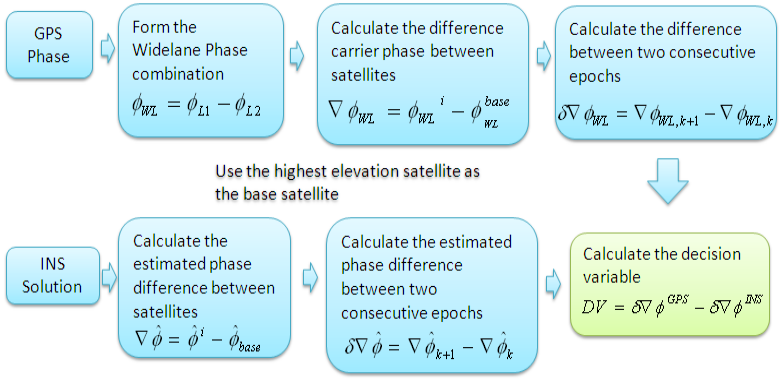
Scheme of WL phase based DV calculation.

The WL phase can be generated by using Equation (3) in cycle units. Apparently it is not a geometry-free combination because it contains the range information. To detect and identify any possible cycle slip, we need a geometry-free quantity, which is called decision variable (DV) in our method. So the INS solutions are used to remove the range information to get a geometry-free combination or the DV:

(3) $$\Phi_{WL}=\Phi_{1}-\Phi_{2}$$

where Φ_1_ and Φ_2_ are the phase measurements in the L1 and L2 frequencies in cycle units, respectively. The procedure of calculation of the DV is shown in Figure 2.

It is worth mentioning that the double differenced algorithm in the proposed method is based on double differencing between two satellites and two consecutive epochs in the time domain. It is therefore not the same as the traditional double difference algorithm in DGPS which is based on double differencing between two receivers and two satellites. The double differenced GPS WL phase observations are generated first. Given satellites' positions, the double differenced INS-derived geometric ranges are computed based on INS positions. Then the DVs are calculated by differencing double differenced GPS WL phase observations and double differenced INS-derived geometric ranges. The WL phase combination has a wavelength of 86 cm, which is much longer than the wavelength on L1 and L2 frequencies. So this can improve robustness and reliability of the testing procedure.

The inter-frequency bias is a delay of the L1 and L2 signals because of different modulations of the L1 and L2 signals. It must be taken into account for any autonomous GPS observation models except for the ionosphere-free combination [25]. Fortunately, this term can be considered to be varied on a daily basis [25,33], so it is safe to assume that they can be cancelled by the differencing algorithm between two consecutive epochs. The receiver clock offset can be removed by the differencing between two satellites, while the satellite clock and orbit errors can be mostly removed by using precise satellite orbit and clock corrections. So the calculated WL phase based DV can be expressed by Equation (4):

(4) $$DV=\delta \nabla \Phi_{WL}-\delta \nabla \Phi_{WL}^{\text{INS}}=-\delta \nabla d_{\text{ion},WL}+\delta \nabla d_{\text{trop}}+\delta \nabla N_{WL}\lambda_{WL}+\delta \nabla \varepsilon_{WL}-\delta \nabla \varepsilon_{\text{INS}}$$

where the symbol δ represents differencing between two consecutive epochs, the symbol ∇ represents differencing between two satellites, *λ_WL_* represents the wavelength of WL phase in the units of meters, *N_WL_* represents the WL ambiguity and it is unitless, *d_ion,WL_* represents the ionospheric delay in WL phase combination in the meter units, *d_trop_* represents the tropospheric delay in meter units, *ε_WL_* represents the WL phase noise in meter units and *ε_INS_* represents the range error of INS-derived geometric range in units of meters.

For the applications when GPS integrates with INS, the GPS data rate is very high, typically 1 Hz or higher, because INS needs frequent updates from GPS to calibrate the inertial sensor biases and to limit the INS error accumulation, so it is reasonable to assume that variations of the ionospheric and the tropospheric delays during such a short time period are small enough to be ignored. Then the WL phase based DV can be simplified as shown in Equation (5). If the errors from double differenced GPS WL phase and INS-derived range are considered to be Gaussian distributed with a mean value of zero, then the DV should also follow the Gaussian distribution with a mean value of zero when the carrier phase measurements are free of cycle slips:

(5) $$DV=\delta \nabla N_{WL}\lambda_{WL}+\delta \nabla \varepsilon_{WL}-\delta \nabla \varepsilon_{\text{INS}}$$

**Figure 3.**
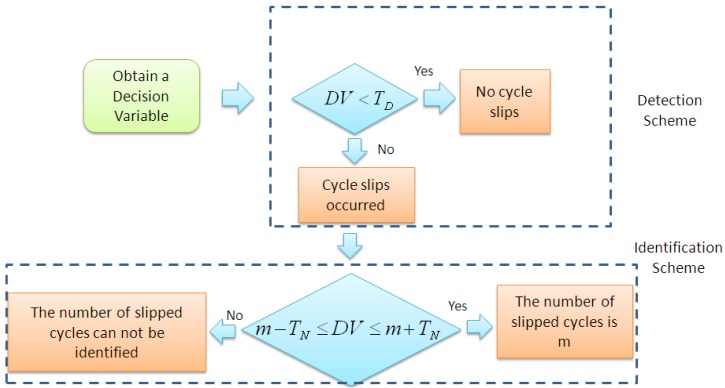
Scheme of cycle slip detection and identification.

Figure 3 shows the scheme of cycle slip detection and identification. *T_D_* and *T_N_* are the thresholds for cycle slip detection and identification, respectively.

Simply speaking, when an estimated DV exceeds a predefined detection threshold, then at least one cycle slip occurred, otherwise no cycle slip is detected. Similarly, if an estimated DV falls into a certain interval determined by an integer number m and a predefined identification threshold, then the number of cycle slips is identified as m. According to [16], there are testing probabilities for false alarm, missed detection, right determination and false determination, which can be described by Equations (6–9), respectively. The false alarm means that the detection threshold is exceeded though no cycle slip occurred, while the missed detection means that the detection threshold is not exceeded though cycle slip is actually present. The right determination means that an estimated DV falls into an interval *I_m_* centered at the number m if indeed m is the number of cycle slips, while the false determination means that an estimated DV lies in a wrong interval *I_k_* leading to a false fixed number of cycle slips. For more details readers can be referred to [3,16,18]:

(6) $$P_{FA}=2\text{erfc}(\frac{T_{D}}{\sigma })$$

(7) $$P_{MD}=\text{erfc}(\frac{m-T_{D}}{\sigma })-\text{erfc}(\frac{m+T_{D}}{\sigma })$$

(8) $$P_{RD}=2\text{erf}(\frac{T_{N}}{\sigma })-1$$

(9) $$P_{FD}=2\sum_{i=1}^{\infty }\left\{\text{erf}(\frac{i+T_{N}}{\sigma })-\text{erf}(\frac{i-T_{N}}{\sigma })\right\}{|}_{i\neq m}$$

where *σ* is the standard deviation of DV in the unit of cycle and m is the number of cycle slips, and $\text{erf}(x)=\frac{2}{\sqrt{\pi }}\int_{0}^{x}e^{-t^{2}}dt$.

As it can be seen from Equations (6–9), the testing probabilities are highly dependent upon the chosen thresholds and the standard deviations (σ) of DV. In practice certain critical testing probabilities like *P_MD_* and *P_FD_* have to be guaranteed and are therefore treated as given values while the thresholds are adjusted according to the estimated standard deviations of DV [16].

### 3.2. EWL Phase Based Cycle Slip Detection and Identification

The EWL phase based cycle slip detection and identification follows the same testing procedure described in Section 3.1. Instead of using the WL phase combination, the EWL phase combination is used in the second step, which can be generated in the unit of cycle as shown in Equation (10). The wavelength of EWL phase is 183 cm, which is 2 times longer than the wavelength of WL phase:

(10) $$\Phi_{\text{EWL}}=4\Phi_{L1}-5\Phi_{L2}$$

### 3.3. Cycle Slip Identification on L1 and L2 Frequencies

The WL and EWL phases are linear combinations of the carrier phase observations on the L1 and L2 frequencies. Based on the identified cycle slips on the WL phase and EWL phase, cycle slips in the L1 and L2 frequencies can be easily determined as shown in Equations (11) and (12):

(11) $$m_{1}=5m_{WL}-m_{\text{EWL}}$$

(12) $$m_{2}=4m_{WL}-m_{\text{EWL}}$$

where *m*_1_ and *m*_2_ represent the number of cycle slips on L1 and L2 frequencies, respectively, *m_WL_* and *m_EWL_* are the number of cycle slips in the WL phase and EWL phase, respectively.

## 4. Field Test Results and Analysis

This section presents the results of two field tests. The field test #1 is an airborne test with a tactical-grade IMU while the field test #2 was conducted based a land vehicle and a consumer-grade MEMS IMU. From the results, we will see that cycle slips can be detected and identified with very high confidence level using both grades of IMUs.

### 4.1. Test Results of Field Test #1

An airborne test has been conducted to verify the performance of a tightly coupled PPP GPS/Tactical-grade IMU system and to evaluate the efficiency of the proposed cycle slip detection and identification algorithm. A SPAN system from NovAtel was mounted in a test airplane and collected both GPS and INS data. The GPS pseudorange, carrier phase and Doppler measurements were collected at 1 Hz and the INS measurements were sampled at 100 Hz.

#### 4.1.1. Navigation Performance of Integrated PPP GPS/Tactical-grade IMU System

**Figure 4.**
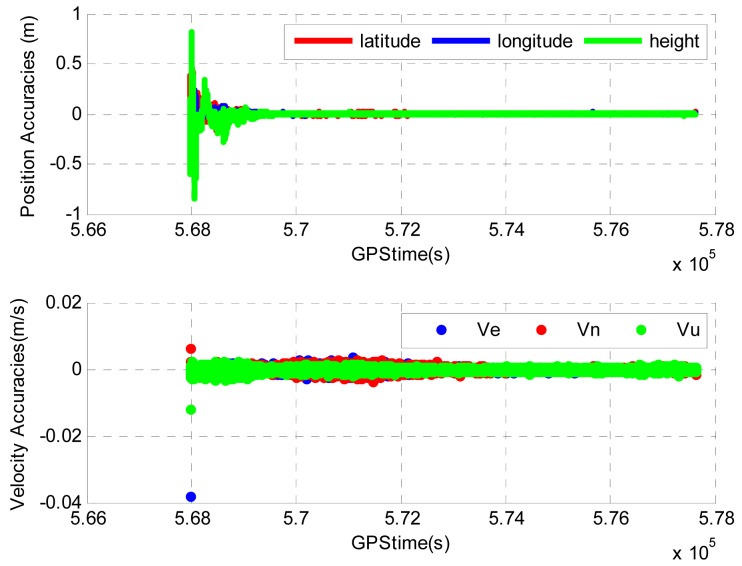
Position and velocity errors of tightly coupled PPP GPS/Tactical-grade IMU system in Field test #1.

To evaluate the position and velocity accuracies of the tightly coupled PPP GPS/Tactical-grade IMU system, a reference solution is obtained by differential GPS/INS processing with a ground base receiver station using a commercial software. The reference solution accuracy is at the level of several centimeters for position and millimeter to centimeter per second for velocity. The difference between the reference and the tightly coupled solutions is given in Figure 4 for both position and velocity.

During the airborne test, the environment for PPP GPS is friendly and the satellite geometry is fairly good. High precision GPS solutions are obtained from the tightly coupled system. The greater position discrepancies at the beginning of the processing as shown in Figure 4 are due to the less accurate position solution from the tightly coupled system. This is because the position solution in PPP GPS requires a time period to converge to centimeter level accuracy due to the fact that the ambiguities are estimated as float numbers and they require a time period to converge to their true values [1,23,34]. This is not the case for velocity estimation, as the velocity solution mainly depends on Doppler measurements.

#### 4.1.2. Efficiency Evaluation of the Algorithm of Inertial Aided Cycle Slip Detection and Identification in Field Test #1

**Figure 5.**
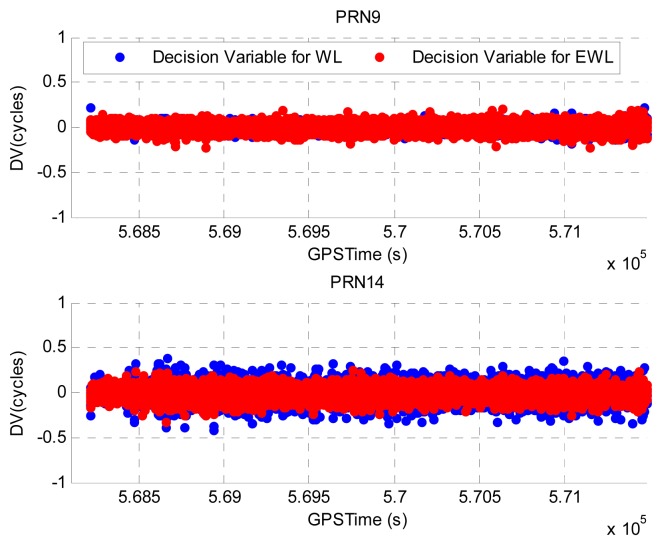
DVs for PRN9 and PRN14 in Field test #1.

Illustrated in Figure 5 are the calculated DVs for PRN9 and PRN14. As GPS data was collected with a data rate of 1 Hz, the interval between two consecutive epochs during which the DVs are calculated is 1 s. The PRN18 has the highest elevation and is selected as the base satellite for the DV calculation. PRN9 has an elevation of about 70° and it represents the high elevation satellites whereas PRN14 has a much lower elevation of about 20° and it represents the low elevation satellites. The blue and red dots represent the computed WL phase based and EWL phase based DVs, respectively. The epochs at which cycle slips are detected have been removed to provide a clear view of the values of DVs. Summarized in Table 1 are the estimated standard deviations (STD) of the DVs for PRN9 and PRN14. Due to the lower elevation, the DVs for PRN14 are noisier and the corresponding STDs are greater comparing to those of PRN9.

As it is difficult to verify the efficiency of the algorithm without knowing the truth, a cycle slip scenario is simulated as shown in Table 2. The threshold of cycle slip detection and identification is set to 0.5 cycles and the corresponding testing probabilities can be calculated based on Equations (6–9). The testing probabilities for PRN9 and PRN14 are listed in Table 3. The FA, MD, RD and FD represent the false alarm, missed detection, right determination and the false determination, respectively. If the calculated probability is smaller than 10^−10^, it will be given as zero, and if it is greater than 0.999999999, it will be given as one. These testing probabilities indicate that cycle slips can be detected and identified with very high confidence level.

**Figure 6.**
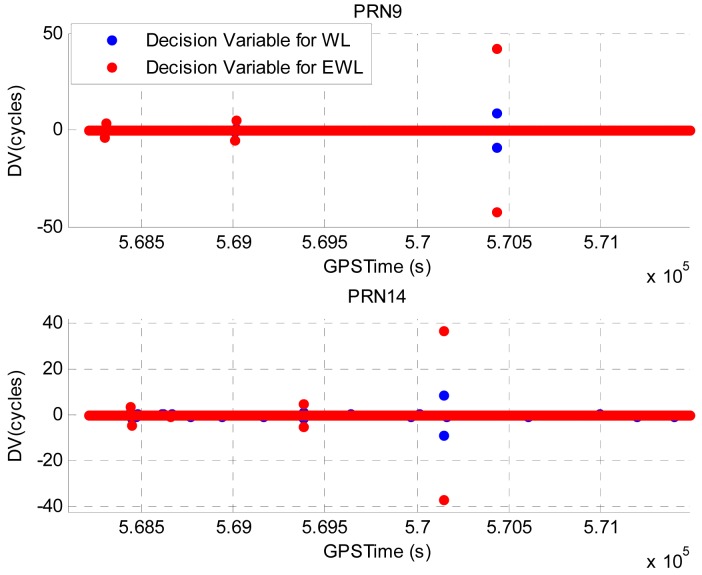
DVs for PRN9 and PRN14 with simulated cycle slips in Field test #1.

Illustrated in Figure 6 are the calculated DVs with simulated cycle slips for PRN9 and PRN14. The great values of DVs are observed at several epochs and they indicate the occurrence of cycle slips. Table 4 summarizes the detected and identified cycle slips using the proposed method. It summarizes the identified cycle slips on WL phase, EWL phase, L1 phase and L2 phase, respectively. Comparing to the simulated cycle slip scenario, all cycle slips have been correctly identified.

After using the simulated cycle slip scenario to verify the proposed cycle slip detection and identification algorithm, we also applied the proposed algorithm to data processing without simulating any cycle slips. As the airborne test was conducted under an open-sky environment (always the case for an airborne test), only very few cycle slips were detected. The improvements on the positioning performance by repairing these cycle slips are very small. More evaluations for the proposed algorithm are conducted in Field test #2 (land vehicle test), which was conducted under half open-sky environment and a significant number of cycle slips occurred due to the signal blockages.

### 4.2. Test Results of Field Test #2

A van test was conducted at a residential area in Calgary, Alberta. A NovAtel OEM4 receiver and a Nav440 MEMS IMU were installed in the vehicle to collect the GPS data and inertial data, respectively. The pseudorange, carrier phase and Doppler measurements were logged with a data rate of 1 Hz in the L1 and L2 frequencies, and the inertial data was collected at 100 Hz. In order to generate an accurate reference solution using a high precision DGPS/INS integrated system, a tactical grade HG1700 IMU was also installed in the vehicle, and another NovAtel OEM4 receiver was set up on a building roof at The University of Calgary to act as a base receiver station. The vehicle stayed still in an open sky environment for about 25 min to let the PPP solution converge before it was driven to a residential area where the GPS signals are frequently blocked by houses and trees beside the road, and then the vehicle was driven in an open sky environment to let PPP solution re-converge. The duration of the whole dataset is about 50 min.

A MATLAB program has been developed which implements the loosely coupled integrated DGPS/INS algorithm and is used to process the DGPS solution and the HG1700 data to generate the reference navigation solution. The DGPS solution is obtained using Waypoint GrafNav 8.10 software with ambiguity integer solution enabled. The dual frequency GPS carrier phase, pseudorange, and Doppler measurements are all used in the data processing. A smoothing algorithm is also employed to further improve the accuracy of the reference solutions. Finally the obtained reference solution is accurate to less than 5 centimeters, which is enough to evaluate the positioning performance of the PPP/INS integrated system. The noise parameters of HG1700 used in the loosely coupled DGPS/INS system are given below in Table 5[35].

#### 4.2.1. Navigation Performance of Integrated PPP GPS/MEMS IMU System

**Figure 7.**
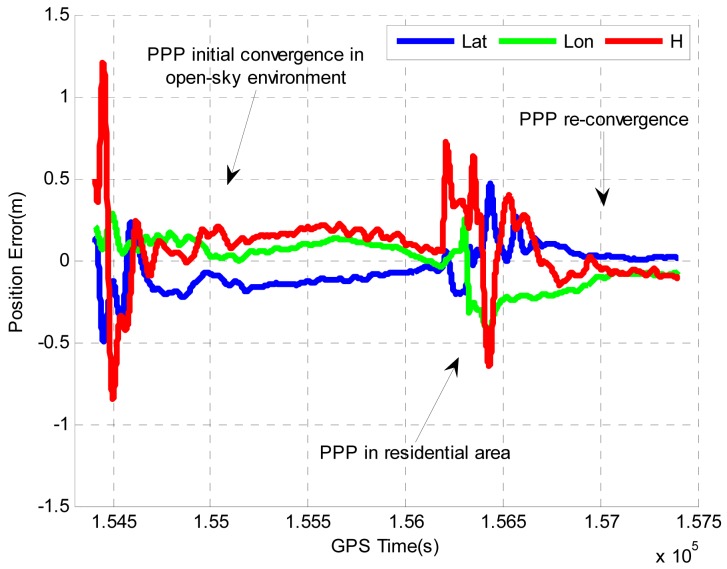
Position and velocity errors of tightly coupled PPP GPS/MEMS IMU system in Field test #2.

Shown in Figure 7 are the tightly coupled PPP GPS/MEMS IMU system position errors. A convergence period is observed before the PPP achieves centimeter to decimeter level accuracy. After the vehicle enters the residential area, there are several significant jumps in the position errors. This is because the signals from low elevation satellites are frequently blocked by houses or trees beside the road, which significantly degrades the satellite availability and geometry. The position solutions start to re-converge to centimeter to decimeter level accuracy after the vehicle left the residential area. A statistical summary of the position errors after the PPP convergence period (and before the vehicle entered the residual area) is summarized in Table 6. The horizontal and vertical RMS position errors are 0.16 m and 0.14 m, respectively.

#### 4.2.2. Efficiency Evaluation of the Algorithm of Inertial Aided Cycle Slip Detection and Identification in Field Test #2

**Figure 8.**
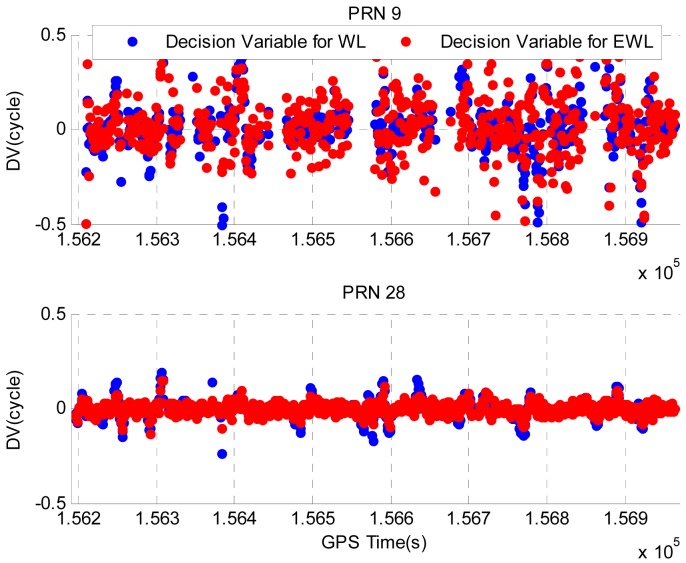
DVs for PRN 9 and PRN 28 in Field test #2.

Illustrated in Figure 8 are the DVs for PRN 9 and PRN 28 during the period when the vehicle was driven in the residential area. The PRN 27 has the highest elevation and is used as the base satellite to compute the DV. The epochs at which cycle slips are detected have been removed to provide a clear view of the DV values. PRN 9 has a lower elevation and it can only be tracked for certain periods of time due to the signal blockages by trees or houses, whereas PRN 28 has a higher elevation angle and it can be tracked continuously during this period. Summarized in Table 7 are the STDs of DVs for PRN 9 and PRN 28. The lower elevation of PRN 9 results the nosier DVs and greater STDs.

Similarly, we use a simulated cycle slip scenario summarized in Table 8 to verify the proposed cycle slip detection and identification algorithm. The threshold of cycle slip detection and identification is set to 0.5 cycles and the testing probabilities for PRN 9 and PRN 28 are listed in Table 9. If the calculated probability is smaller than 10^−10^, it will be given as zero, and if it is greater than 0.999999999, then it will be given as one. Comparing with the testing probabilities obtained in Field test #1, similar values are obtained in Field test #2. This indicates that the use of low-cost MEMS IMU provides similar performance to tactical-grade IMU for the proposed algorithm.

**Figure 9.**
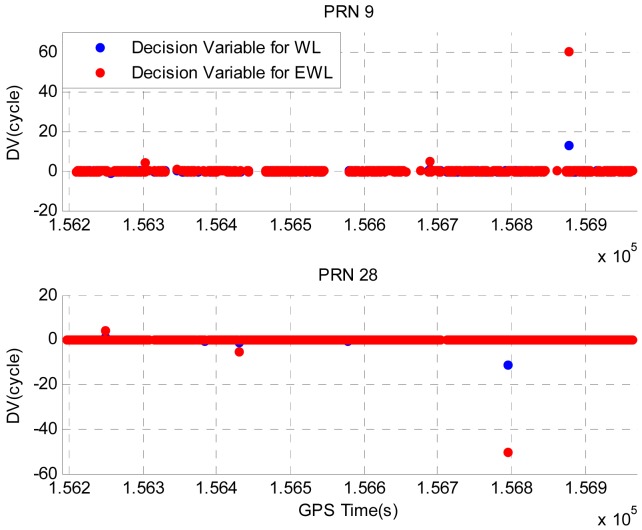
DVs for PRN 9 and PRN 28 with simulated cycle slips in Field test #2.

Illustrated in Figure 9 are the DVs with simulated cycle slips for PRN 9 and PRN 28. As it can be seen, the large values of DVs indicate the occurrence of cycle slips. Table 10 summarized the detected and identified cycle slips using the proposed algorithm. Apparently all cycle slips have been correctly detected and identified.

We also applied the proposed cycle slip detection and identification algorithm to the data processing without simulating any cycle slips to analyze the improvements on the positioning performance. As the vehicle was driven in a residential area, the GPS signals from low elevation satellites were blocked frequently by trees and houses. As a result, a significant number of cycle slips would exist and are expected to be detected. By applying the proposed algorithm, 198 epochs were detected to have cycle slips. The details are shown in Table 11. No cycle slips were detected in the carrier phase measurements for PRN 17 and PRN 28. The results are consistent with the fact that PRN 17 and PRN 28 have higher elevations than other satellites and they can be continuously tracked during the entire period.

**Figure 10.**
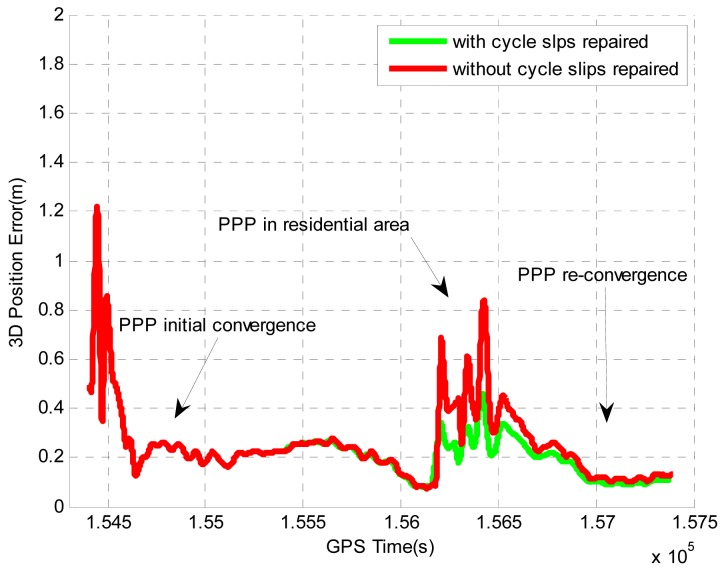
Position accuracy improvement for tightly coupled PPP GPS/MEMS IMU system in Field test #2.

Figure 10 shows the improvements on the position accuracy by repairing the cycle slips using the proposed algorithm. To achieve high level positioning accuracy, the quality control of PPP usually rejects the measurements with large errors (caused by cycle slips or severe multipath).

By using the proposed algorithm, the carrier phase measurements rejected because of cycle slips can be used for the position estimation after repairing the cycle slips. As a result, the positioning accuracy can be substantially improved. Table 12 summarizes the improvements on the position accuracy during the period when the vehicle was driven in the residential area. By repairing the cycle slips, the horizontal and vertical position accuracies are improved by 21.4% and 31%, respectively. Although large improvements are observed on position solutions, the improvements on velocity solutions are very small. This is because the velocity estimation mainly relies on the Doppler measurements, which do not contain any ambiguity term and will not be affected by cycle slips.

## 5. Conclusions

An integrated PPP GPS/INS system can be useful in many applications, such as UAV navigation systems, land vehicle/machine automation and mobile mapping systems. Since carrier phase measurements are the primary observables in PPP GPS, cycle slips, which often occur due to high dynamics, signal obstructions and low satellite elevation, must be detected and repaired in order to ensure the navigation performance. Currently most cycle slip detection and identification methods are designed for DGPS or DGPS/INS systems, which require at least two GPS receivers to implement the observation differencing, and therefore they are not suitable for PPP GPS or integrated PPP GPS/INS systems. Moreover, the methods that use the inertial aiding for cycle slip detection and identification all employ tactical-grade IMUs, which constrains their feasibility for consumer applications due to the high cost of high-end IMUs. Some other methods of cycle slip detection and identification are developed for PPP GPS; however they all have their own limitations as described previously. This paper described a new method of inertial-aided cycle slip detection and identification for the integrated PPP GPS/INS system. With the inertial aiding, it jointly uses the WL and EWL phases to detect and identify cycle slips in the L1 and L2 frequencies. The cycle slips are first detected and identified on the WL and EWL phases with the use of INS solutions, and then cycle slips in the L1 and L2 frequencies will be estimated from the WL and EWL cycle slips. The much longer wavelength of WL and EWL phases will improve the robustness and efficiency of cycle slip detection and identification process even in the presence of residual measurement errors. Two field tests are conducted with a tactical-grade IMU and low-cost MEMS IMU, respectively. During the field tests, the GPS data rate is 1 Hz, which is the most common GPS data rate for the integrated GPS/INS system, as the INS needs frequent update from GPS to calibrate the inertial sensor biases and to limit INS error accumulation. The results indicate that the cycle slips can be detected and identified at very high confidence level with both grades of IMUs. As the INS solutions are essential for the cycle slip detection and identification, the GPS data rate of 1 Hz, or even higher, are highly recommended to ensure the quality of the INS solutions.

## Figures and Tables

**Table 1.** STD of DVs for PRN9 and PRN14 in Field test #1.

**PRN**	**STD for WL (cycle)**	**STD for EWL (cycle)**
9	0.04	0.05
14	0.11	0.08

**Table 2.** Simulated cycle slip scenario in Field test #1.

**Satellite PRN**	**GPS Time (s)**	**Cycle Slip on L1 (cycle)**	**Cycle Slip on L2 (cycle)**
9	568,302	−1	0
	569,010	0	1
	570,430	3	−6
14	568,442	1	0
	569,382	0	−1
	570,140	−4	5

**Table 3.** Statistical testing probabilities for PRN9 and PRN14 in Field test #1.

**PRN**	**WL Phase Combination**			

	**FA**	**MD**	**RD**	**FD**
9	0	0	1	0
14	3e-10	1e-10	1-3e-10	3e-10
	**EWL Phase Combination**			

	**FA**	**MD**	**RD**	**FD**

9	0	0	1	0
14	0	0	1	0

**Table 4.** Identified cycle slips of simulated scenario in Field test #1.

**PRN**	**Time**	**DV/WL**	**DV/EWL**	**CSWL**	**CS/EWL**	**CS/L1**	**CS/L2**

	**Unit: s**	**Unit: cycle**					
9	568,302	−0.99	−3.95	−1	−4	−1	0
	569,010	−0.98	−5.01	−1	−5	0	1
	570,430	8.99	42.01	9	42	3	−6
14	568,442	0.94	3.92	1	4	1	0
	569,382	0.97	4.98	1	5	0	−1
	570,140	−8.90	−37.02	−9	−37	−4	5

**Table 5.** Gauss-Markov parameters for HG1700 IMU.

**Sensor**		**Time Constant**	**Temporal Variance**
**Gyro**	**X**	100 min	0.35 deg^2^/hr^2^
	**Y**	55 min	0.34 deg^2^/hr^2^
	**Z**	84 min	0.47 deg^2^/hr^2^
**Accelerometer**	**X**	170 min	8.0e−8 m^2^/s^4^
	**Y**	68 min	2.5e−7 m^2^/s^4^
	**Z**	152 min	4.8e−7 m^2^/s^4^

**Table 6.** Position accuracy of tightly coupled PPP GPS/MEMS IMU system in Field test #2.

**Lat (m)**	**Lon (m)**	**H (m)**	**Horizontal (m)**	**3D (m)**
0.12	0.09	0.14	0.16	0.21

**Table 7.** STDs of DVs for PRN 9 and PRN 28 in Field test #2.

**PRN**	**STD for WL (cycle)**	**STD for EWL (cycle)**
9	0.13	0.16
28	0.04	0.03

**Table 8.** Simulated cycle slip scenario in Field test #2.

**Satellite PRN**	**GPS Time (s)**	**Cycle Slip on L1 (cycle)**	**Cycle Slip on L2 (cycle)**
9	156,303	1	0
	156,688	0	−1
	156,878	5	−8
28	156,248	1	0
	156,430	0	1
	156,794	−5	6

**Table 9.** Statistical testing probabilities for PRN 9 and PRN 28 in Field test #2.

**PRN**	**Widelane Phase Combination**			

	**FA**	**MD**	**RD**	**FD**
9	5e−8	5e−8	1–1e−7	1e−7
28	0	0	1	0
	**Extra-Widelane Phase Combination**			

	**FA**	**MD**	**RD**	**FD**

9	9−-6	2e−6	1–5e−6	5e−6
28	0	0	1	0

**Table 10.** Identified cycle slips of simulated scenario in Field test #2.

**PRN**	**Time**	**DV/WL**	**DV/EWL**	**CS/WL**	**CS/EWL**	**C/L1**	**CS/L2**

	**Unit: s**	**Unit: cycle**					
9	156,303	1.09	4.07	1	4	1	0
	156,688	1.23	5.12	1	5	0	–1
	156,878	13.10	60.10	13	60	5	–8
28	156,248	1.14	4.08	1	4	1	0
	156,430	–1.03	–5.02	–1	–5	0	1
	156,794	–10.99	–49.98	–11	–50	–5	6

**Table 11.** Cycle slip detection results in Field test #2.

**PRN**	**1**	**4**	**8**	**9**	**11**	**12**	**15**	**17**	**26**	**28**	**Total**
# of Epochs	14	24	78	15	3	13	13	0	38	0	198

**Table 12.** Summary of position accuracy improvements for tightly coupled PPP GPS/MEMS IMU system in Field test #2.

	**Lat (m)**	**Lon (m)**	**H (m)**	**Horizontal (m)**	**3D (m)**
no CS fixed	0.15	0.23	0.29	0.28	0.40
CS fixed	0.12	0.18	0.20	0.22	0.29
Improvement (%)	20.0	21.7	31.0	21.4	27.5
